# Implementing an X-ray validation pipeline for the Protein Data Bank

**DOI:** 10.1107/S0907444911050359

**Published:** 2012-03-16

**Authors:** Swanand Gore, Sameer Velankar, Gerard J. Kleywegt

**Affiliations:** a Protein Data Bank in Europe (PDBe), EMBL–EBI, Wellcome Trust Genome Campus, Hinxton, Cambridge CB10 1SD, England

**Keywords:** validation, data archives, biomacromolecular structure, PDB

## Abstract

There is an increasing realisation that the quality of the biomacromolecular structures deposited in the Protein Data Bank (PDB) archive needs to be assessed critically using established and powerful validation methods. The Worldwide Protein Data Bank (wwPDB) organization has convened several Validation Task Forces (VTFs) to advise on the methods and standards that should be used to validate all of the entries already in the PDB as well as all structures that will be deposited in the future. The recommendations of the X-ray VTF are currently being implemented in a software pipeline. Here, ongoing work on this pipeline is briefly described as well as ways in which validation-related information could be presented to users of structural data.

## Abstract 2

The implementation of a validation pipeline, based on community recommendations, for future depositions of X-ray crystal structures in the Protein Data Bank is described.

## 1. Introduction

The Protein Data Bank (PDB) is the single global repository of experimentally determined three-dimensional structure data on biomacromolecules and their complexes. Since 2003, the PDB has been operated by the Worldwide Protein Data Bank (wwPDB; http://wwpdb.org; Berman *et al.*, 2007 ▶), which consists of the RCSB PDB (Berman *et al.*, 2000 ▶), PDBe (Velankar *et al.*, 2010 ▶, 2011 ▶, 2012 ▶), PDBj (Standley *et al.*, 2008 ▶) and BMRB (Ulrich *et al.*, 2008 ▶). The four partners accept and curate depositions of newly determined structures and the corresponding experimental data and make these available in the PDB archive. They also carry out remediation of the archive, maintain a chemical component database, coordinate the weekly releases of the archive, interact with journals and define and implement procedures and standards for data deposition and annotation. In addition, the wwPDB organization defines policy issues (*e.g.* regarding allowed hold periods and mandatory deposition requirements), validation standards and format specifications, with extensive input from the community (through its advisory board or specially convened task forces).

The structures in the PDB are based on a subjective interpretation of experimental data, which may itself be of variable quality, a process that can lead to errors with varying degrees of impact (Brändén & Jones, 1990 ▶; Morris *et al.*, 1992 ▶; Kleywegt & Jones, 1995 ▶, 1996 ▶, 1997 ▶, 2002 ▶; Hooft *et al.*, 1996 ▶; Kleywegt, 2000 ▶, 2007 ▶, 2009 ▶; Chen *et al.*, 2010 ▶). For this reason, it is crucial to assess the quality and reliability of the resulting models, a process known as validation (Kleywegt, 2000 ▶, 2009 ▶). In the area of protein X-ray crystallography, a wealth of experience has been gained in validation of models, experimental data and the fit of the model to these data. Unfortunately, the application of validation procedures by practising crystallographers has been anything but uniform. In the past decade the amount of structural data archived in the PDB has grown enormously and these data now provide a rich source of information for validation procedures, including examples of various types of errors, a large population sample to estimate *a priori* expectations and test sets for methods development. Simultaneously, a number of unfortunate cases in which published high-profile structures turned out to be seriously flawed (*e.g.* Chang *et al.*, 2006 ▶) have received widespread attention, undermining the confidence of user communities in the reliability of three-dimensional structural data in general (Miller, 2006 ▶, 2007 ▶).

A few years ago, the wwPDB partners realised that there was both a need and an opportunity to consolidate the accumulated experience and expertise in the area of validation of X-ray crystal structures of biomacromolecules. They convened a Validation Task Force (VTF) consisting of experts in the field to provide a set of community consensus recommendations on how to validate X-ray structures upon deposition in the PDB and how to present the results of the validation to the depositor. This wwPDB X-ray VTF (http://wwpdb.org/workshop/2008) has recently published its report with findings and recommendations (Read *et al.*, 2011 ▶). The wwPDB partners have also convened an NMR VTF (http://wwpdb.org/workshop/2010/nmr_validation.html), which is expected to produce an initial set of recommendations early in 2012. PDBe and RCSB PDB, the two organizations who manage the EMDB archive (Lawson *et al.*, 2011 ▶), have also convened a VTF for three-dimensional electron microscopy (3DEM; http://vtf.emdatabank.org/; Henderson *et al.*, 2012 ▶). Additional task forces will inform the wwPDB partners in the future about small-angle scattering techniques and hybrid methods for structure determination.

The goals of structure validation for the PDB are as follows.

(i) To improve the quality and consistency of the structural archive ‘at the gate’. Based on an in-depth report on the quality and quirks of a model, a depositor may identify problems that need further attention, such as limited rebuilding and refinement of a ligand or loop.
(ii) To support editors and referees of papers describing structural data. To this end, the PDB validation reports should contain summary information that compares the quality of a model with that of other models in the archive.
(iii) To help expert and non-expert users of individual released PDB entries to assess their quality and to decide whether the entry is suitable for their needs (*e.g.* docking studies, homology modelling, design of mutants or use as a molecular-replacement probe).
(iv) To help expert and non-expert users to compare a number of related PDB entries in order to identify the model that appears to be of the highest quality among them (Velankar & Kleywegt, 2011 ▶). A typical human protein in the PDB today will occur in several dozen distinct PDB entries: these will be structures determined by different techniques, in different laboratories, under different conditions, with different mutations and with different ligands. Identifying ‘the best’ of these models is a time-consuming challenge for experts and nigh impossible for non-experts at present.
(v) To enable advanced users to identify and reject outliers (in terms of structural quality) when mining or analysing the entire archive or a substantial subset of it.
(vi) To stimulate the adoption of widely accepted state-of-the-art validation methods (and possibly structure-determination protocols) by the community.

Here, we describe our ongoing work on implementing the recommendations of the X-ray VTF (Read *et al.*, 2011 ▶) in a software pipeline. We also describe how validation-related information could be presented to users of structural data. For more general reviews of the types of errors that can occur in protein crystal structures as well as of validation methods that can be used to detect (some of) these, we refer to the literature (Brändén & Jones, 1990 ▶; Kleywegt & Jones, 1995 ▶, 1996 ▶, 1997 ▶, 2002 ▶; Kleywegt, 2000 ▶, 2007 ▶, 2009 ▶).

## 2. A validation pipeline for the PDB

In 2011, the wwPDB X-ray VTF produced a detailed report with recommendations on how to carry out validation of X-ray data, models and the fit of the models to the data (Read *et al.*, 2011 ▶).

Previous checking methods used by wwPDB deposition sites were limited in their scope, *e.g.* Ramachandran analysis (Kleywegt & Jones, 1996 ▶) and comparison of bond lengths and bond angles to the statistics compiled by Engh & Huber (1991 ▶). The checks proposed by the X-ray VTF are more comprehensive and use contemporary methods and underlying distributions. For diffraction data, the recommended checks include assessment of the Wilson plot, identification of outlier reflections, amplitude/intensity mislabelling, anisotropy, twinning, missed symmetry *etc.*; these checks can be carried out with the *phenix.xtriage* program (Adams *et al.*, 2010 ▶). Validation of models should include assessment of the covalent geometry as well as of backbone and side-chain torsion-angle combinations (Ramachandran and rotamer analysis), possible flipping of side chains, van der Waals overlaps using a model that includes (riding) H atoms, un­satisfied hydrogen-bond donors and acceptors *etc.* These checks can be carried out with *MolProbity* (Chen *et al.*, 2010 ▶) and *WHAT_CHECK* (Hooft *et al.*, 1996 ▶). For assessing the agreement between the model and data, the VTF recommends the use of *R* and *R*_free_ (Brünger, 1992 ▶) as global parameters and per-residue assessment of the real-space *R* value (RSR; Jones *et al.*, 1991 ▶) by calculating RSR-*Z* scores as is performed by the Uppsala Electron-Density Server (EDS; Kleywegt *et al.*, 2004 ▶). Some of the statistics will need to be calculated or aggregated per residue, per chain or for the whole entry (*e.g.* individual Ramachandran outliers are important, but also the percentage of outliers for each individual protein chain and for the entry as a whole).

**Figure 1:**
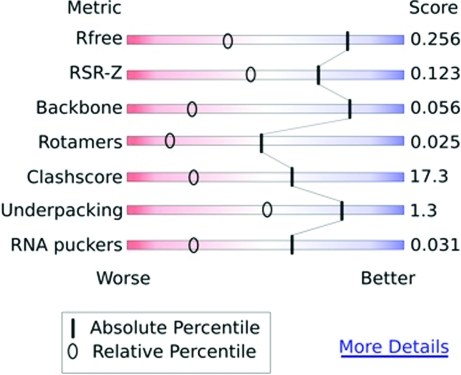
Mock-up of a slider graph designed to convey information about key validation criteria for an existing PDB entry or a new deposition. Absolute percentile scores reflect how well the structure scores on the corresponding criteria compared with all PDB entries. Relative percentile scores show how it compares with structures determined at similar resolution. (Figure kindly provided by Jane Richardson.)

To facilitate interpretation of the quality scores and comparison of an entry with other structures, the X-ray VTF recommends calculating percentile ranks for a number of key statistics. The advantage of this is that users would not need to know what the various statistics represent or what the ‘raw’ values mean. The percentile scores could be relative to the entire archive (*i.e.* compared with all other X-ray structures in the PDB) or to a subset of entries (*e.g.* compared with the 1000 X-ray structures with the most similar resolution). The former would be most useful for users of PDB data and the latter for depositors themselves as well as for journal editors and referees. The VTF recommends summarizing the percentile scores on some key criteria using sliders (Fig. 1 ▶).

The VTF also made several recommendations about the way in which the results of the validation procedure could be presented. After the validation has been carried out, a human-readable (PDF) report should be produced that contains information that helps non-experts assess the quality and alerts experts (in particular, the depositor) to unusual features that may require further refinement, rebuilding or verification. In addition, a machine-readable file should be produced that can be used by graphics software to guide model analysis and rebuilding, and that can be loaded into a database and used to drive services that report and visualize validation-related information to the wider user community once a PDB entry has been released.

Currently, the wwPDB partners are developing a completely new software system for deposition and annotation of structural data that, once operational, will be used by all sites. Validation pipelines for X-ray, NMR and EM models and data will form an integral part of this new system. The implementation of the X-ray validation pipeline is being carried out at the Protein Data Bank in Europe (PDBe; http://pdbe.org). At a later stage, the validation pipelines will also be made available as anonymous web servers so that experimentalists will be able to assess the quality of their models prior to deposition.

**Figure 2:**
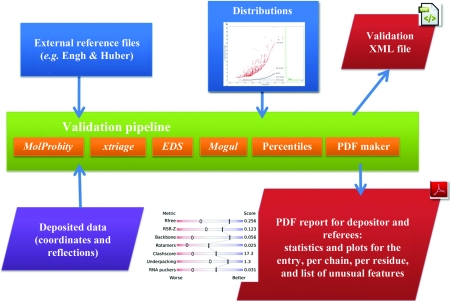
Overview of the components, input and output of the first version of the wwPDB X-ray validation pipeline that is currently being implemented following the recommendations of the wwPDB X-ray Validation Task Force. In future versions of the pipeline, additional validation modules will be included, *e.g.* *WHAT_CHECK*.

For practical reasons, the development of the X-ray validation pipeline is an incremental process. In the first version, it will include *phenix.xtriage* (Zwart *et al.*, 2005 ▶) and components of the EDS software (Kleywegt *et al.*, 2004 ▶) to validate the structure-factor data and the fit of the model to the data. The protein and nucleic acid components of the model itself will be validated using components of *MolProbity* (Chen *et al.*, 2010 ▶) and *WHAT_CHECK* (Hooft *et al.*, 1996 ▶). Finally, the geometrical quality of ligand molecules will be assessed using the program *Mogul* (Bruno *et al.*, 2004 ▶), which will be provided by the Cambridge Crystallographic Data Centre (CCDC; http://www.ccdc.cam.ac.uk/products/csd_system/mogul). An overview of the major components and input and output of the pipeline is shown in Fig. 2 ▶.

The implementation of the X-ray validation pipeline is carried out for each of the component modules in turn. Initially, the contributed software is left intact as much as possible, with the input provided in the expected formats (*e.g.* PDB and MTZ files rather than the native mmCIF format that is used by the new joint deposition and annotation system) and the output filtered to extract the relevant information. Auxiliary software is developed as needed, *e.g.* to calculate distributions and percentile ranks and to generate a PDF report from the raw machine-readable validation-results file. In some cases, methods will have to be modified or developed. For example, RSR-*Z* score calculations as carried out by EDS rely on average and standard deviation values for the common amino-acid and nucleotide residues in different resolution shells (Kleywegt *et al.*, 2004 ▶). Since ligands often occur only once or a few times in the PDB, no statistically meaningful distribution is available for their RSR values. However, ligands could be grouped based on the number of non-H atoms that they contain and whether or not they contain ‘non-­pharmaceutical’ atoms. Average RSR values and standard deviations could then be calculated in resolution shells for entire groups of ligands of similar size and chemistry. We are currently exploring the feasibility and effectiveness of this novel approach.

The first priority for our work on the X-ray validation pipeline is to integrate it into the new wwPDB deposition and annotation system and to implement it on the hardware of the wwPDB partner sites. Once this has been achieved, we will endeavour to make the pipeline available as a separate web-based server as well. This would allow crystallographers to assess the quality of intermediate models using the same criteria that will be used upon deposition of the final model, and pinpoint any parts or aspects of the model that require further attention.

A number of practical decisions will also have to be made. Clearly, percentile ranks will change as more entries are deposited in the PDB archive. For practical reasons, we intend to produce a versioned PDB-wide list of validation statistics annually, which will then be used to calculate the percentile ranks for a year until the next version is released. These files, as well as XML files with validation data for all released PDB entries, will be made publicly available so that they can be used by external software and database developers.

It is anticipated that the wwPDB X-ray VTF will reassess the state-of-the-art in validation methodology occasionally (*e.g.* every five years) and adjust or augment its recommendations accordingly.

The wwPDB partners hope that many journals will follow the lead of the IUCr journals and begin to require submission of the PDB validation report whenever a manuscript describing a new biomacromolecular structure is submitted for publication.

## 3. Presenting validation-related information to users

The wwPDB partners engage in friendly competition with regard to the presentation of data from the PDB archive to users and the development of value-added services and resources. Hence, they are free to and will independently develop methods to use the validation data for released entries and present it to users.

**Figure 3:**
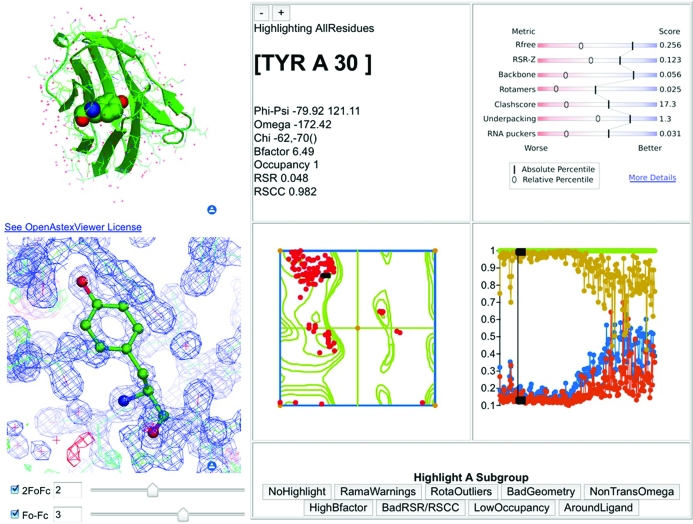
Design mock-up of the user interface of an enhanced version of EDS currently under development at PDBe. This resource will present data (both overall and per-residue) calculated by the wwPDB X-ray validation pipeline as well as interactive displays of models and electron-density maps. All the panels are linked so that if residues are selected in one panel they will be highlighted in all other panels as well. The buttons in the lower right corner can be used to select subsets of residues, *e.g.* all Ramachandran plot outliers or all residues in the binding site of a certain ligand.

As described previously (Velankar & Kleywegt, 2011 ▶), PDBe intends to assimilate the EDS functionality and integrate it into its data infrastructure. The functionality of EDS will be enhanced by adding data produced by the wwPDB validation pipeline to provide a comprehensive analysis of the quality and reliability of crystal structures. Fig. 3 ▶ shows a mock-up of what such a resource could look like for a released PDB entry. The EDS software has been re-implemented and will provide electron-density maps; the wwPDB validation facility described above will provide a host of additional quality information. In the current design plans, the service will display linked views of the model and electron-density maps as well as one-dimensional plots (*e.g.* RSR-*Z* scores per residue) and two-dimensional graphs (*e.g.* Ramachandran plot) and an information panel. Whenever a residue is selected in any of the views or graphs, it will become active in all others as well. There will also be a mechanism to select ‘interesting’ subsets of residues, *e.g.* residues in a ligand-binding site or all Ramachandran outliers.

Finally, as and when the recommendations of the NMR and 3DEM VTFs are implemented, PDBe will also develop services to facilitate validation and analysis of the models produced by these techniques. A first glimpse of what could be performed with respect to analysis and validation of NMR entries is available as a PDBe service called Vivaldi (http://pdbe.org/vivaldi; Velankar *et al.*, 2012 ▶).

## 4. Concluding remarks

As the various subdisciplines of molecular and cellular structural biology mature, we expect that a consensus about sensible and informative validation methods will emerge. It took the field of protein X-ray crystallography some 25 years to go through this, at times painful, process. In the mid-1980s it was first realised that crystallographic models could occasionally be significantly in error (Brändén & Jones, 1990 ▶); now, the community has finally agreed that deposition of models and data, as well as validation of both, should be mandatory for every new structure that is archived in the PDB.

It is the professional obligation of every structural biologist to produce the best possible models that are supported by their experimental data and to teach their students and colleagues how to achieve this (Brändén & Jones, 1990 ▶; Kleywegt, 2000 ▶; Kleywegt *et al.*, 2004 ▶; Rupp, 2010 ▶). In addition, it is important that the community as a whole promotes a basic understanding among non-experts of how structures come about, the fact that sometimes errors are made and how validation methods can help to pinpoint problems in individual models and enable users to select the most appropriate model.

The wwPDB partners are committed to utilizing established validation methods to improve the quality and integrity of the archive and to enabling users of structural data to make informed choices about the most suitable models for their purposes, without requiring them to become experts in any structure-determination method or even in validation method­ology.
